# Interfacial solar evaporator for brine treatment: the importance of resilience to high salinity

**DOI:** 10.1093/nsr/nwab118

**Published:** 2021-07-01

**Authors:** Baoxia Mi

**Affiliations:** Department of Civil and Environmental Engineering, University of California, Berkeley, USA

As reverse osmosis (RO) technologies have improved, desalination has become a viable approach to addressing water scarcity, by treating various alternative water resources such as seawater, brackish water and wastewater [1]. However, the management of brines has emerged as one of the key issues preventing desalination from being further adopted, especially in locations where use of ocean outfalls or deep well injection is impractical. To advance desalination in these places, zero-liquid-discharge (ZLD) is often desired to achieve the ultimate separation of water and solid. However, current approaches to achieving ZLD are either land intensive—as is the case with evaporation ponds—or energy intensive—requiring multiple steps such as brine concentrators and crystallizers to concentrate and eventually crystallize the brine stream [2]. RO membranes are not suitable for treating high salinity waters, when the salt content is typically higher than 7.5 wt%, due to the demand of high operation pressure and membrane fouling. Therefore, high salinity is often the root challenge for brine treatment, causing fouling/scaling and high energy demand.

The pioneering work by Xu *et al.* presented an innovative process that offers a means of concentrating brines and recovering salts using only solar energy [3]. The process used a system of self-assembled particles made of an expanded polystyrene (EPS) core and graphene oxide (GO) shell to introduce interfacial solar evaporation that enabled complete water-solute separation. Interfacial solar evaporation has been widely studied in recent years as a sustainable technology with regard to addressing water-energy challenges [4]. The photothermal, adsorptive, charge, lamellar and multifunctional properties of GO have made it a popular material for potential application in desalination [5,6]. GO was also used as a solar absorber to coat various 2D supports for interfacial solar evaporation of brines [7,8]. Compared to previous work, the approach reported by Xu *et al.* has several advantages [3].

**Figure 1.**
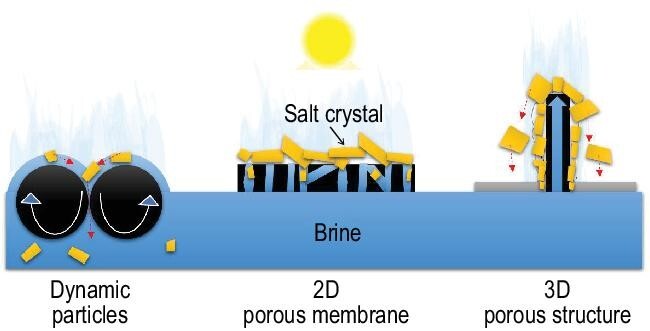
Illustration of the production and accumulation of salt crystals in three different interfacial solar evaporation systems: self-assembled dynamic particles, 2D porous membranes and 3D porous structures.

First, the dynamic nature of the solar evaporator enabled by a sheet of self-assembled EPS-GO particles offered higher evaporative water flux at high salinity than 2D membrane evaporators. For example, Xu *et al.* reported a 12% decrease in evaporative water flux when the salt content increased from 0 to 20 wt% [3]. In a similar study using GO to make a 2D membrane evaporator, the evaporative water flux decreased by 75% when the salt content increased from 0 to 15 wt% [7]. This was because the 2D membrane evaporators relied on the water transport through the porous membrane structure to supply water for evaporation, while the pores are likely to get clogged by salt crystals during the evaporation (Fig. 1). The dynamic particle evaporator, however, got water supply by surface tension across the particle surface. So, there was no pore clogging effects at high or saturated salt content.

Second, in the dynamic particle system [3], the particle aggregation driven by surface tension together with the subsequent particle rotation driven by salt production on the top generated a self-cleaning mechanism to prevent salt accumulation on the surface. As shown in Fig. 1, this offered substantial advantages over 2D membrane evaporators that typically had the accumulation of salt crystals on the surface [7]. Although the accumulation of salt did not further decrease evaporative water flux, surface scrubbing was needed to remove the salt when substantial accumulation occurred. Another potential strategy of introducing the self-cleaning mechanism is to employ a 3D interfacial solar evaporator. As illustrated in Fig. 1, the 3D evaporator will allow the salt crystals to fall off under gravity. An additional benefit of the 3D evaporator is that it may help reduce the footprint of the evaporation pond by increasing the total evaporation surface area or the evaporation area index (EAI), which is defined by the ratio of the total evaporation area to the projected ground area.

Additionally, the self-aggregation property of the particle system is convenient for enabling large-scale application [3]. Nevertheless, with regard to further advancing interfacial solar evaporation into a technology that would have broad application to brine management, there are still issues that need to be addressed. One is the effects of brine composition (i.e. brine components other than NaCl). When the technology is used to treat complex brines, it is possible that certain minerals (e.g. Ca^2+^, Ba^2+^, SO_4_^2−^, silica) and organic matter may lead to fouling that compromises the evaporation performance. Another is the potential of resource recovery from brines. Brines may contain trace elements (Cr, Se) that could decrease the value of the recovered salts or major species (e.g. Ca, PO_4_^3−^) that could be valorized. Therefore, selective separation may be needed to maximize the cost benefit of the technology.

In summary, interfacial solar evaporation has the potential to transform the current approach to solving the brine management challenge. Although it is quite possible that gradual improvements in high-pressure RO membranes and crystallizers will reduce the cost and energy consumption associated with ZLD and near-ZLD technologies, interfacial solar evaporation technology has a unique potential to transform existing brine management facilities. Starting with processes that already have brine evaporation ponds, interfacial solar evaporation technology is easily retrofitted to existing ponds to accelerate evaporation, resulting in huge economic savings compared to the current practice.

***Conflict of interest statement*.** None declared.
